# Three Memoirs on the Development and Structure of the Teeth and Epithelium

**Published:** 1841-12

**Authors:** 


*Three Memoirs on the Development and Structure of the Teeth and Epithelium.* Read at the ninth Annual Meeting of the British Association for the encouragement of science, held at Birmingham, in August, 1839; with diagrams exhibited in illustration of them. BY ALEXANDER NASMYTH, F. L. S., F. G. S., Member of the Royal College of Surgeons, &c. LONDON, JOHN CHURCHILL, 8vo. pp. 47, 1841.

WHEN we saw the publication of these memoirs announced, in the "*Boston Medical and Surgical Journal,*" we were of the opinion that they constituted the treatise upon the development, structure and diseases of the teeth, promised by the author about two years ago, and in our last number spoke of it as such. In this, we were disappointed,—the long expected work is yet to come. Mr. Nasmyth is not only an able writer, but he is a close investigator, and his researches in this department of minute structural anatomy, have thrown much new and valuable light upon it.

We should be glad to notice the foregoing memoirs at length, but having only just received them, our time will not at present permit us to do so. We, however, copy the following, which will enable the reader to form an idea of Mr. Nasmyth's peculiar views on the structure of the teeth, taken from the *London Lancet*, from the "Boston Medical and Surgical Journal."
*Balt. Ed.*

"Our readers are aware that a tooth consists of three substances: of *enamel*, which forms a thin crust over the crown; of *ivory*, or tooth bone, which constitutes the chief bulk of the tooth; and of *cementum*, or crusta petrosa, which invests the root of the tooth, and under certain circumstances forms a thin lining to, or completely fills up the cavitas pulpæ. The ivory or tooth-bone consists of fibre-like, undulating tubuli, which traverse a dense, interfibrous or intertubular substance. The intertubular substance has been described hitherto by our best anatomists, among whom may be named Perkinje, Retzius, and Muller, as uniform and structureless. But Mr. Nasmyth is "disposed to believe that it is not only organized, but differently and characteristically so in different animals, as to be capable of affording valuable aid to the naturalist in classifying the animal kingdom." According to Mr. Nasmyth, the producing structure of the ivory, viz., the pulp, "is cellular throughout its entire structure;" the producing structure of the enamel, viz., the internal surface of the capsule, is also cellular; and the ivory and the enamel which are formed by a transformation of the pulp, and of the internal surface of the capsule, bear distinct traces of the cellular texture of which their basis is composed.

This important discovery, the originality of which cannot, we believe, be questioned, was made by Mr. Nasmyth in the inverse order to that which we have adopted in describing its nature.

"My attention," the author observes, "was first drawn to the structure of the interfibrous substance on examining a delicate section of the fossil tooth of a rhinoceros, by the aid of a very high magnifying power, of one-tenth of an inch focal distance, and of the most perfect kind, with an achromatic condenser of light. The instrument with which I have conducted my researches, and upon the accuracy of which I place the greatest reliance, is that of Mr. Powell. In the section of the tooth of the rhinoceros to which I have just alluded, will be observed an appearance of cells or compartments;" an appearance which the author's subsequent investigations proved to be universal both in fossil and in recent teeth.

Mr. Nasmyth has also made researches into the structure and composition of the tubuli, which that gentleman terms "fibres," of the teeth of different animals. These he finds to

"Present an interrupted or baccated appearance, as if they were made up of different compartments—an obvious concomitant of the cellular structure of the interfibrous material. The size and relative position of these portions or divisions of a fibre differ in various series of animals. In the human subject, for instance, each compartment of the fibre is of an oval shape, and its long, small extremity is in apposition with that next adjoining. The long axis of the oval corresponds with the course of the fibre. In some species of the monkey tribe, the fibre appears to be composed of two rows of compartments parallel to each other. In the orang outang the fibre is composed of rhomboidal divisions, and in the baboon they are oval, like those of the human subject, and the surfaces of the long axes are in apposition. In fact, each class of animals seem to have a distinct characteristic appearance, but all are similar in respect to the general baccated appearance."

Of the application of his views to practical purposes, the author remarks:—

"All systems of dental structure which have hitherto been propounded have failed, I think, to explain facts of daily occurrence; but they may be accounted for, I venture to assert, by the cellular organization of the interfibrous substance which has been improperly termed structureless, and by the peculiar baccated arrangement of the fibres.

On the structure of the enamel, we read the following:—

"According to the views of Retzius, Perkinje, and the recent investigators of the structure of the teeth by the aid of the microscope, the enamel consists of fibres, running in a direction from the centre to the circumference of the tooth. On making a section of the enamel in a direction parallel to the transverse diameter of the tooth, the appearance as described by these writers is observed, and they are said to be seen to terminate in a hexagonal form beneath the investing crusta petrosa. If, however, a different section of the enamel of the human tooth be made, for instance, one near the surface, parallel to the vertical direction or long axis of the tooth, an appearance presents itself which has induced me to take a different view of the nature of the structure of the enamel. The section of the enamel presents compartments or divisions, but of a different character from those I have already spoken of as existing in the interfibrous structure of the ivory. Each compartment of the enamel is of a semicircular form, and the convexity of the semicircle or arch looks upward towards the free external portion of the tooth."

